# Vitamin D/VDR signaling pathway ameliorates 2,4,6-trinitrobenzene sulfonic acid-induced colitis by inhibiting intestinal epithelial apoptosis

**DOI:** 10.3892/ijmm.2015.2150

**Published:** 2015-03-20

**Authors:** TONG ZHU, TIAN-JING LIU, YONG-YAN SHI, QUN ZHAO

**Affiliations:** 1 Department of Pediatric Orthopedics, Shengjing Hospital of China Medical University, Shenyang, Liaoning 110004, P.R. China; 2 Key Laboratory of Health Ministry for Congenital Malformation, Shengjing Hospital of China Medical University, Shenyang, Liaoning 110004, P.R. China; 3 Department of Pediatrics, Shengjing Hospital of China Medical University, Shenyang, Liaoning 110004, P.R. China

**Keywords:** vitamin D, vitamin D receptor, 2, 4, 6-trinitrobenzene sulfonic acid, inflammatory bowel disease, p53 upregulated modulator of apoptosis, apoptosis

## Abstract

Increasing epidemiological data have suggested a link between vitamin D deficiency and the incidence of inflammatory bowel disease (IBD). In the present study, we confirmed that vitamin D deficiency, as well as the decreased local expression of vitamin D receptor (VDR), was prevalent in an IBD cohort. The excessive apoptosis of intestinal epithelial cells (IECs) partly accounts for the development of colonic inflammation and eventually results in IBD. Based on the established inhibitory effects of the vitamin D/VDR pathway on IEC apoptosis, we treated mice with 2,4,6-trinitrobenzene sulfonic acid (TNBS)-induced colitis with paricalcitol, a vitamin D analog, in order to investigate the mechanisms responsible for the inhibitory effects of the vitamin D/VDR pathway. We observed that following treatment with vitamin D, the mice presented with only minor bodyweight loss, and the mice also showed improved histological scores and decreased intestinal epithelial permeability compared with the vehicle-treated group. The colonic mRNA expression of inflammatory cytokines and chemokines was markedly suppressed, indicating less severe colitis in the vitamin D-treated mice. Subsequently, we investigated p53 upregulated modulator of apoptosis (PUMA) and p53, two major independent pathways of apoptosis, as well as caspase-3. We found that the vitamin D-treated mice had lower expression levels of caspase-3 than the vehicle-treated mice. PUMA expression showed the same tendency; however, the p53 protein level was not altered. The present study indicates that vitamin D attenuates the development of TNBS-induced colitis by inhibiting the apoptosis of IECs. The mechanisms involved include the downregulation of PUMA expression. Our data provide experimental support for the clinical trials of vitamin D intervention in patients with IBD.

## Introduction

Crohn’s disease (CD) and ulcerative colitis (UC) are the two major forms of chronic inflammatory bowel disease (IBD). Increasing epidemiological data have suggested a link between vitamin D deficiency and the incidence of IBD, and vitamin D deficiency has been shown to be prevalent in patients with IBD (1–3). As the vitamin D receptor (VDR) is highly expressed in intestinal epithelial cells (IECs), the vitamin D/VDR signaling pathway may play a key role in the pathogenesis of IBD. Moreover, it has been reported that VDR gene polymorphisms are associated with the incidence of IBD (4,5) and VDR knockout mice have been shown to have a compromised mucosal barrier, leading to increased susceptibility to mucosal damage and an increased risk of developing IBD (6). These data suggest that vitamin D and/or VDR serve as an environmental and/or genetic factor in the pathogenesis of IBD.

The intestinal epithelial barrier plays an important role in the development of colitis, which consists of a monolayer of epithelial cells and intercellular junctions between adjacent cells that seal the paracellular gap (7). The barrier regulates intestinal permeability between the lumen and mucosal layer, protecting mucosal immune cells from coming into contact with gut microbiome, harmful solutes, toxins and luminal antigens (8–11). The compromise or disruption of the intestinal barrier function results in intestinal inflammation. The integrity of the intestinal epithelial barrier is preserved by thousands of IECs. The aberrant apoptosis of IECs has been proven to be a major pathophysiological mechanism of increased gut permeability and inflammation (12–15). Previous studies have reported increased IEC apoptosis in patients with UC and CD, as well as in murine models of colitis (12–15). The excessive apoptosis of IECs causes the disruption of the epithelial barrier, leading to increased intestinal permeability and the subsequent invasion of pro-inflammatory substances (13–15). Those substances can induce the production of inflammatory cytokines and chemokines in the colonic lamina propria. This vicious series of intestinal events promotes the development of colonic inflammation and eventually results in IBD.

1,25-Dihydroxyvitamin D_3_ (calcitriol) is the active form of vitamin D and binds with VDR. Apart from its classical calcium-regulating effect, vitamin D serves as a potent regulator of multiple biological activities, including antimicrobial activities, the inhibition of apoptosis and immunomodulatory functions (16). Recently, it was suggested that VDR transgenic mice exhibit less colitis than wild-type mice, indicating the protective role of VDR in the development of intestinal inflammation (13). Thus, we hypothesized that treatment with vitamin D may attenuate the severity of 2,4,6-trinitrobenzene sulfonic acid (TNBS)-induced colitis by inhibiting IEC apoptosis.

## Materials and methods

### Human biopsies

Human biopsies were collected through endoscopic examination from patients with UC and CD at the Shengjing Hospital of China Medical University between January 2012 and December 2012. Non-IBD control subjects were selected from subjects who underwent endoscopic examination for the elimination of other bowel diseases and were proven to be free of gut-related diseases. Written informed consent was obtained from each subject prior to enrollment in the study.

### Induction of acute colitis

Male adult C57BL6/J mice, weighing 20–25 g, were supplied by the Center of Experimental Animals of China Medical University, Shenyang, China. All animal procedures were reviewed and approved by the Laboratory Animal Ethics Committee of China Medical University. The mice were anaesthetized by an intraperitoneal (i.p.) injection of cocktail anesthetics [ketamine (ketavest 100 mg/ml); Pfizer, New York, NY, USA) and [xylazine (Rompun 2%); Bayer HealthCare, Leverkusen, Germany). The mice were treated with 100 mg/kg TNBS (Sigma-Aldrich, St. Louis, MO, USA) dissolved in 50% ethanol by intrarectal injection with an 18-gauge stainless steel gavage needle. The control group was treated with 50% ethanol without TNBS, as previoulsy described (17).

### Treatment with vitamin D or the vehicle

The TNBS and control groups were randomly divided into 2 groups, respectively. One group was treated with the vitamin D analog, paricalcitol (Sigma-Aldrich), dissolved in propylene glycol:ethanol, 90:10 at 0.5 *μ*g/kg body weight, while the other group was administered the dissolvent only. Paricalcitol or the vehicle were administered through i.p. injection 30 min before, 1, 3 and 5 days after the TNBS injection.

### Reverse transcription-quantitative PCR (RT-qPCR)

The mice were sacrificed 48 h after the TNBS injection. A 2-cm section of the colon was cut from each mouse and the colonic mucosa was harvested. Total RNA was isolated using TRIzol reagent (Invitrogen, Carlsbad, CA, USA). First-strand cDNA was synthesized from 3 *μ*g of total RNA in a 20 *μ*l reaction system using M-MLV reverse transcriptase (Invitrogen) and random primers. Quantitative (real-time) PCR (qPCR) was performed on a Roche 480 Real-Time PCR system using SYBR-Green PCR reagent kits (Clontech Laboratories Inc., Mountain View, CA, USA). The relative amounts of mRNA were calculated using the 2^−ΔΔCt^ formula. β-2 microglobulin (B2M) was used as an internal control. The sequences of the primers used for PCR are provided in Table I.

### Western blot analysis

The colonic mucosa lysates were separated by SDS-PAGE, and the proteins were transferred electrophoretically onto polyvinylidene difluoride membranes (Millipore, Billerica, MA, USA). We used ImageJ software (National Institutes of Health, Bethesda, MD, USA) to quantify the density of the bands normalized to that of β-actin. The antibodies used in the present study included: anti-VDR (C20; 1:2,000; Santa Cruz Biotechnology Inc., Santa Cruz, CA, USA), anti-β-actin (A5316; 1:10,000; Sigma-Aldrich), anti-p53 (9282; 1:3,000; Cell Signaling Technology, Beverly, MA, USA), anti-p53 upregulated modulator of apoptosis (PUMA; 7467; 1:3,000; Cell Signaling Technology) and anti-caspase 3 (9662; 1:1,000; Cell Signaling Technology) antibodies.

### Hematoxylin and eosin staining

The whole colons were harvested on day 4 after the TNBS injection. The colon morphology was recorded and scored according to a macroscopic scoring system (18). The distal colon were fixed overnight with 4% formaldehyde in PBS (pH 7.4), dehydrated with graded alcohol, placed in xylene for 1 h and then embedded in paraffin at 60°C. Sections of the colon (4 *μ*m) were stained with hematoxylin and eosin. Colon sections from all the groups were examined for any histological changes. From each section, 20 random spots were examined under a microscope (Leica DFC425; Leica Microsystems, Heerbrugg, Switzerland) at a magnification of x100. Microscopic scoring was performed for each spot according to a scoring system (18) independently by two pathologists who were blinded to the group design.

### Measurement of serum 25-hydroxyvitamin D

The human serum 25-hydroxyvitamin D concentration (nmol/l) was measured using a commercial 25-hydroxyvitamin D EIA kit (Immunodiagnostic Systems PLC, Boldon, Tyne & Wear, UK) according to the manufacturer’s instructions.

### FITC-dextran intestinal permeability

The mice were denied access to food, but were allowed to drink water for 4 h before gavage. FITC-4 kDa dextran (50 mg/ml) (Sigma-Aldrich) was administered by gavage at a dose of 4 *μ*l/g body weight through an 18-guage stainless steel gavage needle. Blood serum was collected 3 h later. The blood serum was then placed at 150 *μ*l/well into a 96-well plate and analyzed using a Synergy HT microplate reader (BioTek Instruments, Inc., Winooski, VT, USA).

## Results

### Low 25-hydroxyvitamin D levels and reduced VDR expression in patients with UC and CD compared to the normal controls

**Figure 1:**
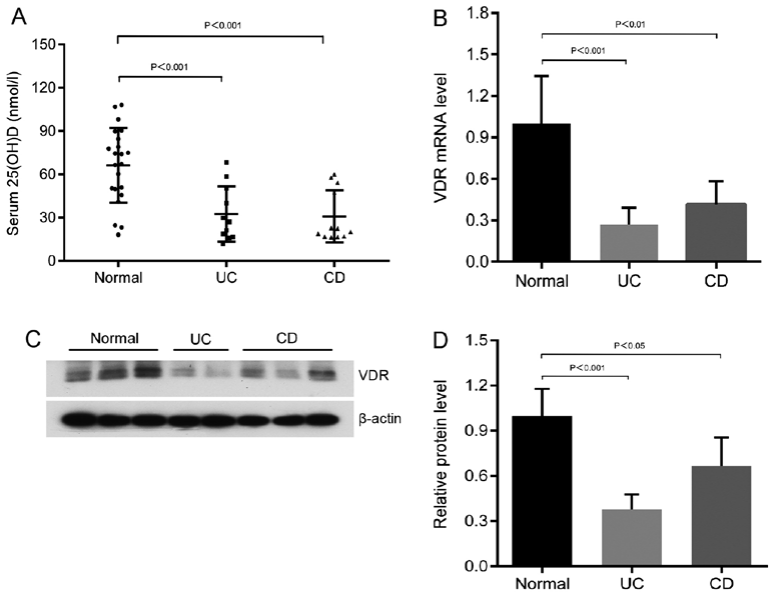
Low vitamin Ds level and reduced VDR expression in patients with UC and CD compared to the normal controls. (A) Serum 25(OH)D concentrations in normal controls and patients with IBD. (B) Relative expression of VDR in normal controls and patients with IBD analyzed by RT-qPCR. (C) Representative western blots of colonic biopsies from normal controls and patients with IBD. (D) Densitometric quantification of VDR expression compared to β-actin. Values are presented as the means ± SD. UC, ulcerative colitis; CD, Crohn’s disease (n>10 in each group). VDR, vitamin D receptor; IBD, inflammatory bowel disease; 25(OH)D, 25-hydroxyvitamin D.

The average serum 25-hydroxyvitamin D levels in the patients with UC and CD were significantly lower than those of the normal controls (Fig. 1A). The 25-hydroxyvitamin D levels of the majority of patients with IBD were in the vitamin D deficiency range (<50 nmol/l; Fig. 1A). In addition, the mRNA expression of VDR was markedly decreased in the patients with UC and CD compared to the normal subjects (n>6; Fig. 1B). Western blot analyses with anti-VDR antibody revealed the decreased expression of the colonic VDR protein level in the patients with UC and CD (Fig. 1C and D). These data from human subjects indicate that a low vitamin D and VDR protein expression may be a pathological factor for the development of colitis.

### Administration of vitamin D ameliorates TNBS-induced colitis

**Figure 2:**
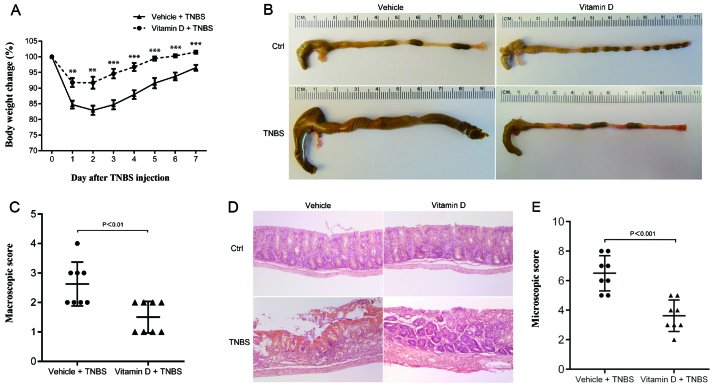
The vitamin D analog, paricalcitol, ameliorates TNBS-induced colitis. (A) Body weight changes over time in the vehicle-treated group and vitamin D-treated group after the TNBS injection. ^**^P<0.01, ^***^P<0.001 vs. vehicle treatment (n=8 in each group). (B) Colon morphology. (C) Macroscopic scoring of colon morphology. (D) H&E staining of distal colon. (E) Microscopic scoring of H&E-stained slides (original magnification, x100) from the vehicle- and paricalcitol-treated groups on day 4 after the TNBS injection or from the control group (50% ethanol injection). TNBS, 2,4,6-trinitrobenzene sulfonic acid.

We used a mouse model of TNBS-induced colitis to mimic the pathological process of IBD. Paricalcitol, a vitamin D analog, was administrated to investigate the protective role of the vitamin D/VDR signaling pathway. Paricalcitol has been proven to exert the same curative effect as calcitriol, but it produces less side-effects, including less hypercalcemia (19,20). After the TNBS injection, the body weight of the mice in both the vitamin D-treated group (VD) and the vehicle-treated group (VE) decreased over time, but the mice in the VD group only developed mild colitis with minor weight loss (Fig. 2A). In the gross observation, the colons of the mice in the VD group were much closer to normal than those of the mice in the VE group (Fig. 2B). Microscopic examination revealed severe hemorrhage, inflammatory cell infiltration and the breakdown of the normal intestinal tissue barrier in the mice in the VE group, while the mice in the VD group presented minimal histological damage (Fig. 2D). The mice in the VE group also had a higher macroscopic score (Fig. 2C) and histological score (Fig. 2E) than the mice in the VD group. These results provide evidence that TNBS-induced colitis may be substantially suppressed by the administration of vitamin D.

### Administration of vitamin D downregulates the expression of pro-inflammatory cytokines and chemokines

**Figure 3:**
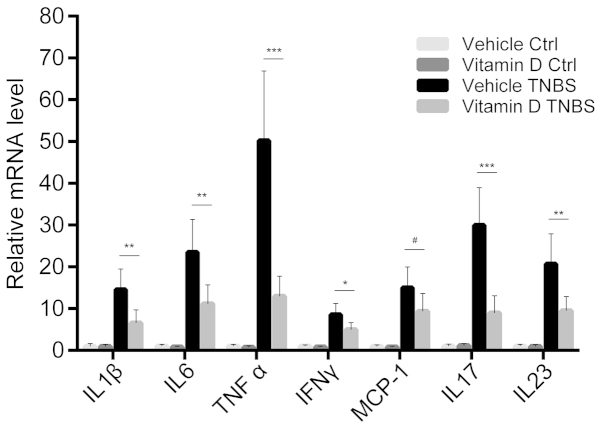
RT-qPCR quantification of pro-inflammatory cytokines and chemokines in the colon mucosa from the vehicle- and paricalcitol-treated mice on day 2 after the TNBS injection or in the control group (50% ethanol injection). ^#^P>0.05, ^*^P<0.05, ^**^P<0.01, ^***^P<0.001 (n=6-8 in each group). TNBS, 2,4,6-trinitrobenzene sulfonic acid.

We further examined the expression of pro-inflammatory cytokines and chemokines in the colonic mucosa. The mRNA expression of cytokines and chemokines was markedly increased by the TNBS injection, while paricalcitol markedly reversed this increase in the expression of the majority of cytokines. This tendency was most obvious with the expression of tumor necrosis factor (TNF)-α and interleukin (IL)-17, two important cytokines associated with the Th1 and Th17 response, respectively. However, monocyte chemotactic protein-1 (MCP-1) was the only chemokine which showed no statistically significant differences in its expression between the VD and VE group (Fig. 3). These result prove that the administration of vitamin D has a direct inhibitory effect on the inflammatory status during the development of colitis.

### Administration of vitamin D protects against barrier disruption and inhibits the increase in intestinal pe rmeability

**Figure 4:**
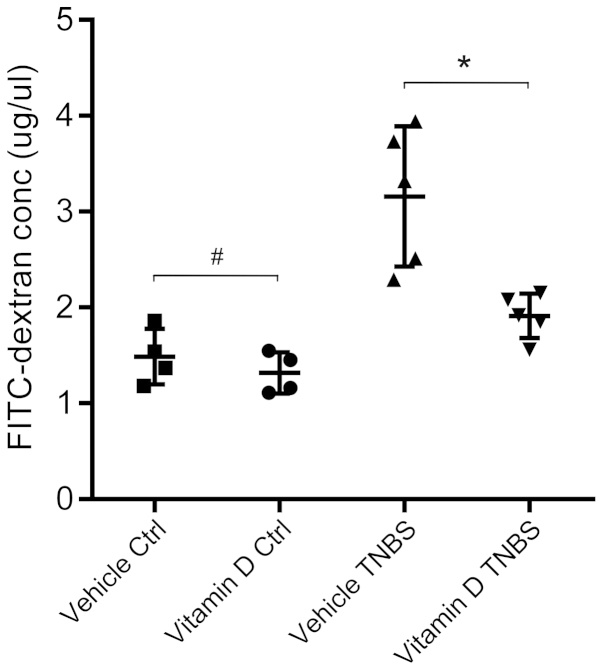
Paricalcitol decreases colon mucosa permeability after the TNBS injection. ^#^P>0.05, ^*^P<0.05 (n=4–5 in each group). TNBS, 2,4,6-trinitrobenzene sulfonic acid; conc, concentration.

We evaluated intestinal permeability following the administration of vitamin D in our mouse model of TNBS-induced colitis. After the TNBS injection, the permeability of the intestinal barrier increased in both the VD and VE group, while the serum concentration of FITC-4-kDa dextran in the mice in the VD group was exclusively lower than that of the mice in the VE group. In other words, the administration of vitamin D protects the integrity of the intestinal barrier and, thus, inhibits the increase in intestinal permeability (Fig. 4).

### Administration of vitamin D inhibits intestinal epithelial apoptosis by suppressing the induction of the PUMA apoptotic pathway

**Figure 5:**
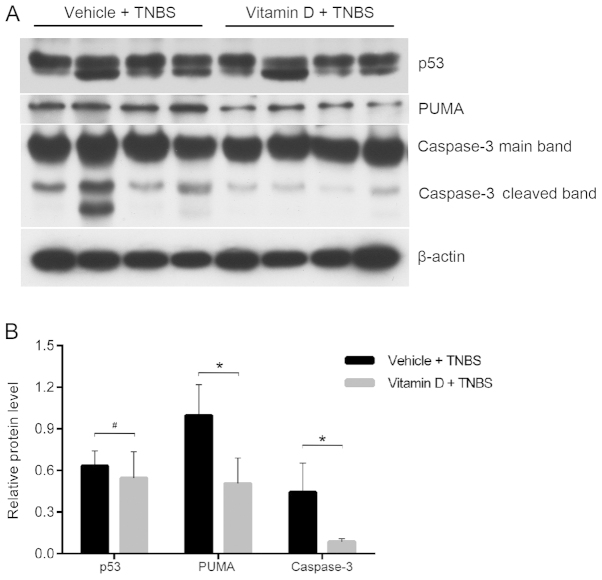
Vitamin D supplementation attenuates colon epithelial cell apoptosis. (A) Western blot analysis and (B) densitometric quantification of the colonic mucosal apoptotic proteins, p53, p53-upregulated modulator of apoptosis (PUMA) and caspase-3, in the vehicle- and paricalcitol-treated mice after the TNBS injection on day 2. ^#^P>0.05, ^*^P<0.05 (n=6 in each group). TNBS, 2,4,6-trinitrobenzene sulfonic acid.

In order to further disclose the mechanisms responsible for the protective effects of vitamin D, we analyzed the expression of p53 and PUMA, two upstream pro-apoptotic proteins of caspase-3. They independently mediate the apoptosis of IECs in patients and mice with colitis (15). Using western blot analysis, we found that the cleavage of caspase-3 was markedly attenuated by treatment with paricalcitol (Fig. 5A). PUMA expression was also decreased in the mice in the VD group; however, no differences were observed in the p53 protein level (Fig. 5). These findings suggest that enhanced vitamin D/VDR signaling inhibits IEC apoptosis by downregulating PUMA expression.

## Discussion

Epidemiological evidence suggests a link between vitamin D deficiency and an increased risk of developing IBD (1–3). The incidence of IBD is parallel to exposure to sunshine, with the highest incidence around the north pole and the lowest along the equator (21,22). Other studies have suggested a link between VDR gene polymorphisms and the risk of developing IBD (4,5). *Taq*I, *Bsm*I, *Fok*I and *Apa*I are the four VDR polymorphisms associated with the higher incidence of IBD, despite variability among different races and populations (4,5,23). Based on the population of northeast China in the present study, we obtained a similar finding, namely that patients with IBD had a worse vitamin D status and lower VDR expression than the normal controls. These data confirm the role of the vitamin D/VDR pathway in the development of gut inflammation, and provide valuable insight into the genetic therapy of IBD.

VDR is highly expressed in the intestine. The classical function of VDR in the small intestine is to regulate the transportation and absorption of calcium and maintain calcium homeostasis. However, the function of VDR in the colon remains to be illustrated. Kong *et al* (6) first reported that vitamin D deficiency compromises the mucosal barrier, leading to increased susceptibility to mucosal damage and an increased risk of developing IBD. Recently, another study suggested that epithelial VDR signaling plays an important role in the homeostasis of luminal microorganisms, antigens and the body (13). VDR is also expressed in immune cells (24). The endogenous serum metabolite of vitamin D, calcitriol, is considered a true steroid hormone, and similar to other glucocorticoids and gonadal hormones, may exert several immunomodulatory effects (24–26). Accumulating evidence indicates an important role of vitamin D in reducing the risk of developing several chronic inflammatory or autoimmune conditions, such as multiple sclerosis, type 1 diabetes and rheumatoid arthritis (24–26). Moreover, vitamin D/VDR pathway dysfunction has been shown to promote the development of inflammation in IL-10 knockout mice, a model of IBD (27). These laboratory data provide a therapeutic foundation for enhancing vitamin D/VDR signaling to inhibit intestinal inflammation.

Clinical studies have revealed that vitamin D supplementation can deter the pathological process of IBD and relieve the symptoms (reviewed in 28); however, the mechanisms responsible for this effect have not yet been fully elucidated. In this study, we found that the vitamin D analog, paricalcitol, substantially alleviated the severity of colitis induced by TNBS, a model of Th1-mediated colitis. The effects of paricalcitol were, at least in part, mediated through the inhibition of the apoptosis of IECs.

PUMA is a key mediator of IEC apoptosis in IBD (15). PUMA is a pro-apoptotic Bcl-2 family member that interacts with anti-apoptotic Bcl-2 family members to activate Bax and/or Bak. This activation induces mitochondrial apoptosis and eventually leads to cell death through the caspase cascade (29–31). Excess epithelial cell apoptosis causes the focal disruption of the intestinal mucosal barrier, leading to the invasion of luminal pathogens and increased intestinal permeability (6). This study demonstrated that the vitamin D analog, paricalcitol, inhibited the activation of PUMA in IECs after the TNBS injection, and therefore, maintained the integrity of the intestinal epithelial barrier. An enhanced epithelial barrier can prevent luminal microorganisms and antigens from invading. In this way, it attenuates the release of pro-inflammatory cytokines and chemokines and relieves inflammatory responses in the colon. This may be one of the pivotal mechanisms through which vitamin D inhibits the development of intestinal inflammation.

In conclusion, this study provides evidence that vitamin D attenuates the development of colitis by inhibiting the apoptosis of IECs. The mechanisms involved include the downregulation of PUMA expression. The present study may shed new light on the curative mechanisms of vitamin D in patients with IBD.

## Figures and Tables

**Table I:** Primers used in this study for PCR.

Primer name	Forward (5′→3′)	Reverse (3′→5′)
Human VDR	ACCTGGTCAGTTACAGCATC	ACTGACGCGGTACTTGTAGT
Human B2M	TGGGTTTCATCCATCCGACA	ACGGCAGGCATACTCATCTT
Mouse IL-1β	AATGAAAGACGGCACACCCA	TGCTTGTGAGGTGCTGATGT
Mouse IL-6	CCTCTGGTCTTCTGGAGTACC	ACTCCTTCTGTGACTCCAGC
Mouse TNF-α	ATGAGCACAGAAAGCATGA	AGTAGACAGAAGAGCGTGGT
Mouse IFN-γ	TTCTTCAGCAACAGCAAGGC	TCAGCAGCGACTCCTTTTCC
Mouse MCP-1	GCTCAGCCAGATGCAGTTAA	TCTTGAGCTTGGTGACAAAAACT
Mouse IL-17	TCTCCACCGCAATGAAGACC	CACACCCACCAGCATCTTCT
Mouse IL-23 p19	GCTGTGCCTAGGAGTAGCAG	TGGCTGTTGTCCTTGAGTCC
Mouse B2M	CGGCCTGTATGCTATCCAGA	GGGTGAATTCAGTGTGAGCC

VDR, vitamin D receptor; B2M, β2 microglobulin; IL, interleukin; TNF, tumor necrosis factor; MCP-1, monocyte chemoattractant protein-1.
